# Akathisia rate among psychiatric patients treated with Clozapine compared to Risperidone.

**DOI:** 10.1192/j.eurpsy.2023.2298

**Published:** 2023-07-19

**Authors:** R. Stryjer, A. Shelef

**Affiliations:** 1 Abarbanel Mental Health Center, Bat Yam; 2 Tel Aviv University, Tel Aviv, Israel

## Abstract

**Introduction:**

Patients’ cooperation, especially in medication taking, constitutes a significant challenge in psychiatric care. Psychiatric patients are often reluctant to take a specific pharmacological treatment because of side effects. Akathisia and other extra pyramidal symptoms are side effects that could result into real danger and are thought to be the main reasons of discontinuing pharmacological treatment. Despite this, some gaps in research knowledge exist on the rate of psychiatric patients that suffer from Akathisia. For example, Clozapine treatment, despite its efficacy, is often disqualified due to side effects concern. The research literature barely refers to the development of extrapyramidal symptoms, and more specifically Akathisia. There are few reports about Akathisia when taking Clozapine. It results into a lack of objective studies and knowledge based only on subjective clinical evaluations.

**Objectives:**

This study aims at comparing Clozapine and Risperidone in general, with or without polypharmacy and more especially, in terms of Akathisia.

**Methods:**

This research was carried out in Abarbanel Mental Health Center over a period of six months. The study population included 100 patients, including hospitalized and ambulatory patients. They were equally divided into two groups: 50 patients who received Clozapine, and 50 patients who received Risperidone. It should be noted that due to polypharmacy, 17 patients in the Clozapine group and 6 patients in the Risperidone group received additional anti-psychotic treatment. Akathisia was evaluated using Barnes Akathisia Scale (BAS).

**Results:**

The demographic and clinical data was found identical in both groups. We found that Akathisia symptoms appeared 1.4 times more in patients who took Risperidone only, compared to those who took Clozapine only (p < 0.05). This research emphasizes the irrelevance of existing data on anti-psychotics’ treatments, while 24% of the patients treated with Clozapine and 34% of the patients treated with Risperidone showed Akathisia symptoms.

**Conclusions:**

Akathisia symptoms are very common in patients who take anti-psychotic treatment as Clozapine and Risperidone. Previous research revealed that Akathisia is very common for Risperidone patients, however the symptoms are often neglected in patients taking Clozapine. We show in our research that the rate of Akathisia is high in both treatments. Therefore, the importance of diagnosing akathisia should be emphasized in patients treated with Clozapine. Moreover, Akathisia should receive much more importance when choosing anti-psychotic treatment.

**Disclosure of Interest:**

None Declared

